# p*K*_50_—A Rigorous Indicator of Individual Functional Group Acidity/Basicity in Multiprotic Compounds

**DOI:** 10.1021/acs.jcim.3c00187

**Published:** 2023-04-27

**Authors:** Robert Fraczkiewicz, Marvin Waldman

**Affiliations:** Simulations Plus, Inc., 42505 10th Street West, Lancaster, California 93534, United States

## Abstract

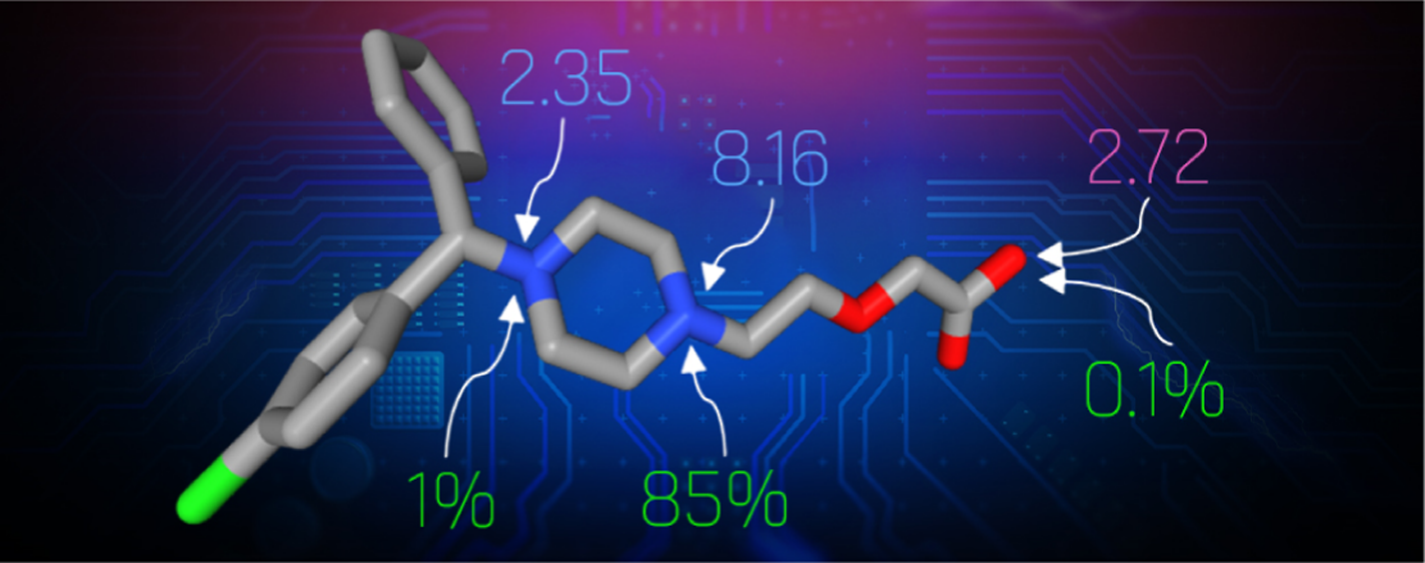

In this work, we show that the apparent p*K*_a_ measured by standard titration experiments is an insufficient measure of acidity or basicity of organic functional groups in multiprotic compounds—a frequent aspect of lead optimization in pharmaceutical research. We show that the use of the apparent p*K*_a_ in this context may result in costly mistakes. To properly represent the group’s true acidity/basicity, we propose p*K*_50_—a single-proton midpoint measure derived from a statistical thermodynamics treatment of multiprotic ionization. We show that p*K*_50_, which may be directly measured in specialized NMR titration experiments, is superior in tracking the functional group’s acidity/basicity across congeneric series of related compounds and converges to the well familiar ionization constant in the monoprotic case.

## Introduction

Protic ionization strongly influences properties and behavior of chemical compounds dissolved in polar solvents. Of these, water is the most important solvent in a number of sciences, such as biochemistry, pharmaceutical science, medicinal chemistry, ecology, toxicology, and agrochemistry. The level of interest in ionization phenomena can be judged by the large number of publications on this topic in the recent scientific literature. Long gone are the times when most molecules of interest were monoprotic in which a single ionization site provided unambiguous interpretation of p*K*_a_ data and a well-defined “acid” or “base” label. Modern molecules are mostly multiprotic, featuring complex dissociation patterns. The underlying issues have been summarized and illustrated in recent publications.^1−4^ The reader is encouraged to review these first, since not all the details are discussed below.

**Figure 1:**
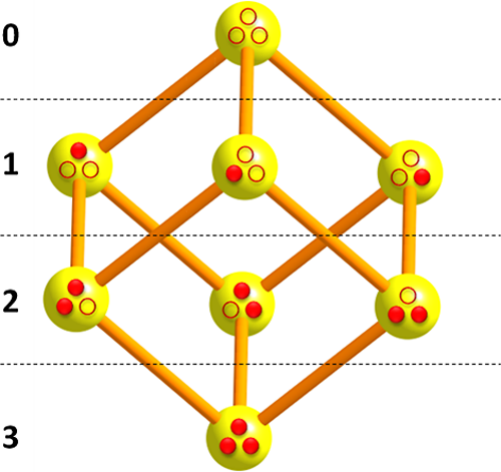
Schematic representation of protonation microstates of a molecule with three ionizable sites. Full and empty circles indicate protonated (1) and unprotonated (0) sites, respectively. Bars symbolize reversible one-proton transitions. Numbers to the left are the total number of bound protons (i.e., the protonation macrostate).

In general, a compound with *n* ionization sites exists in solution as a collection of 2^*n*^ ionization states called microstates or microspecies. Each pair of related microstates differing by one proton is joined by an elementary chemical equilibrium characterized by an ionization microconstant. This observation was noted in the early 20th century,^5^ has received a solid theoretical treatment,^6−8^ and has been confirmed by numerous experiments.^9^ It is crucial to have a clear intuitive understanding of the microstate distribution. The “2^*n*^” number results from all possible combinations of the n sites being either protonated or deprotonated. Each of the microstates can be represented by a binary vector of length *n*, e.g., (0, 0, 1, 0, 1, 1, ..., 1), where 1 and 0 indicate the presence and absence of a proton at a given ionization site, respectively. In the case of *n* = 3, the associated eight microstates (0,0,0), (0,0,1), ..., (1,1,1) can be depicted as the 3D vertices of a cube connected by 12 edges (i.e., 1-proton transitions, Figure 1).

In the more general case, microstates of an *n*-protic molecule can be portrayed as vertices of a hypercube in *n* dimensions. The number of hypercube vertices is 2^*n*^, and its number of edges is *n*2^*n*–1^. For *n* = 10, the number of microstates is equal to a staggering value of 1024! Thus, one can see why, in general, a complete picture of microscopic ionization (i.e., the determination of all microconstants) for complex multiprotic molecules is virtually impossible to obtain experimentally.^8^

**Figure 2:**
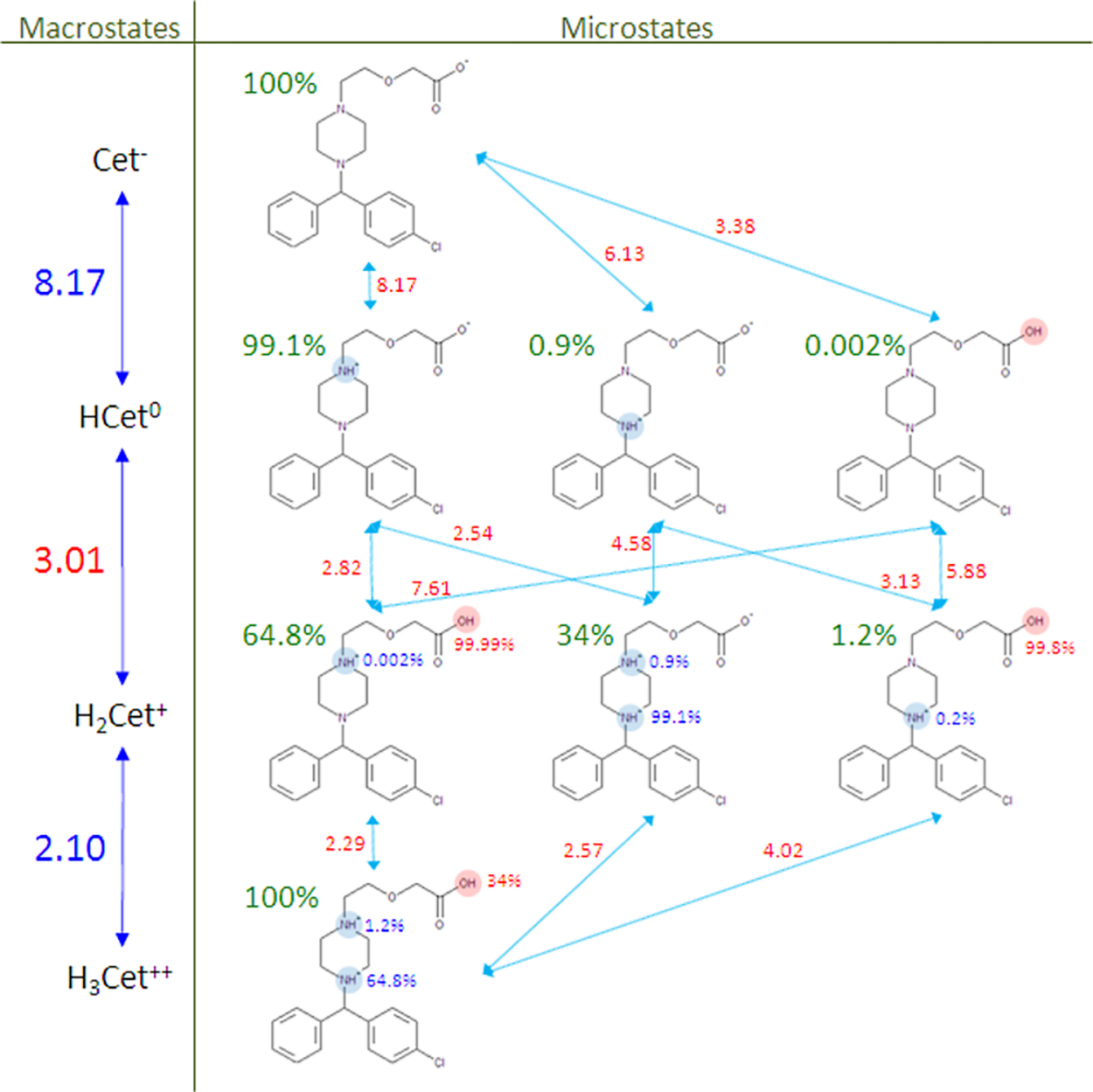
Detailed view of the dissociation macro- (in the left column) and microstates (in the right column) compiled from the recently published results measured for cetirizine—a triprotic molecule denoted symbolically by “Cet”.^9^ Individual microstates have been arranged in a way corresponding to Figure 1. Green percentage numbers next to microstates indicate relative contribution (probability) of this microstate to its parent macrostate. Red numbers next to reaction arrows indicate p*K*_a_ microconstants. Numbers next to protonated groups indicate dissociation probability of that group in the given substrate microstate.

Standard titration experiments are able to resolve only the global formal charge states of the molecule in question, i.e., the macrostates yielding no more than *n* of the usually reported apparent p*K*_a_ constants (macroconstants). Resolution of the microstates requires the use of highly specialized experimental techniques, normally not performed in routine work.^6−13^ One of the reports studied aqueous ionization of cetirizine (an antihistamine drug sold in the USA under the trade name ZYRTEC) as its subject.^9^ We used numerical results of this impressive work, and in Figure 2, we present all the ionization microstates of cetirizine arranged analogously to the scheme depicted in Figure 1.^a^

**Figure 3:**
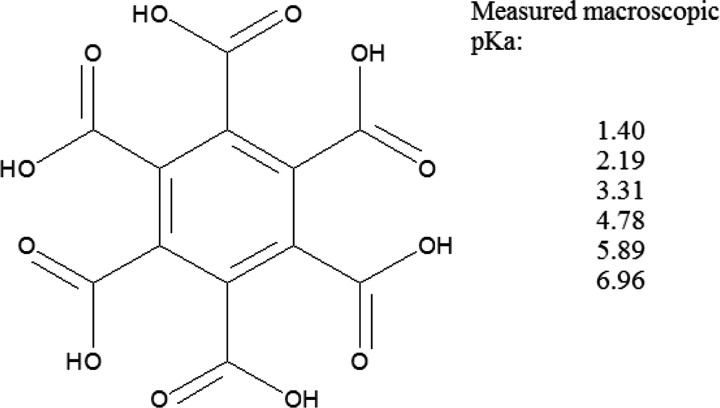
Macroscopic p*K*_a_ measured for mellitic acid.^14^Supporting Information 1 contains ionization microstates.

It is apparent that the knowledge of all the microconstants is the source of very rich and detailed information about all the protonation microstates, such as their relative contributions to the respective parent macrostates as well as, for a given microstate, relative probability of dissociation for each of their attached protons. For example, the neutral macrostate labeled by the zero formal charge (HCet^0^) is strongly dominated (99.1%) by a zwitterionic microstate with one proton bound to the piperazine nitrogen distal to the aromatic rings. No such dominance exists, however, for the +1 macrostate where not one but two microstates make nontrivial contributions. By reviewing the published experimental data, it is evident that the case of multiple microstates with comparable contributions happens more often than expected. The occurrence of this phenomenon naturally increases with the number of ionizable groups. Therefore, the prevalent view of multiprotic ionization occurring sequentially (i.e., one ionizable group at a time) is generally incorrect, even in the approximate sense. The observed macroconstants, 2.10, 3.01, and 8.17, are complex mathematical functions of the relevant microconstants^8^ and are assignable only to the formal charge states of the cetirizine molecule acting as the substrate, i.e., H_3_Cet^++^, H_2_Cet^+^, and HCet^0^, respectively. Attempts to “assign” each of these numbers to an individual ionizable group are doomed to failure. Although one can argue that the 8.17 macroconstant approximately “belongs” to the distal nitrogen due to the strong dominance of one particular microstate, a similar argument fails for the other two macroconstants, as evident from Figure 2. Stark evidence further supporting this truth comes from an impressive experimental work of Maxwell and Partington^14^ who measured all six ionization macroconstants for mellitic acid (Figure 3).

These p*K*_a_ values range from 1.4 to almost 7, spanning 5.6 orders of magnitude and resulting, naturally, from complex interactions between 64 of the acid’s microstates (see Supporting Information 1).^b^ “Assigning” these different p*K*_a_ values to individual carboxyl groups is obviously nonsense since they are all equivalent! The above considerations lead us to the following important conclusion:

In general, macroscopic p*K*_a_ values obtained by standard titration for multiprotic molecules are not properties of individual ionizable groups.

Thus, we have arrived at the central problem addressed by this article: if routinely measured macroscopic p*K*_a_ values are not properties of individual groups, then how can one discern group contributions to multiprotic ionization? We next propose new concepts that successfully address this question.

## Theory

**Figure 4:**
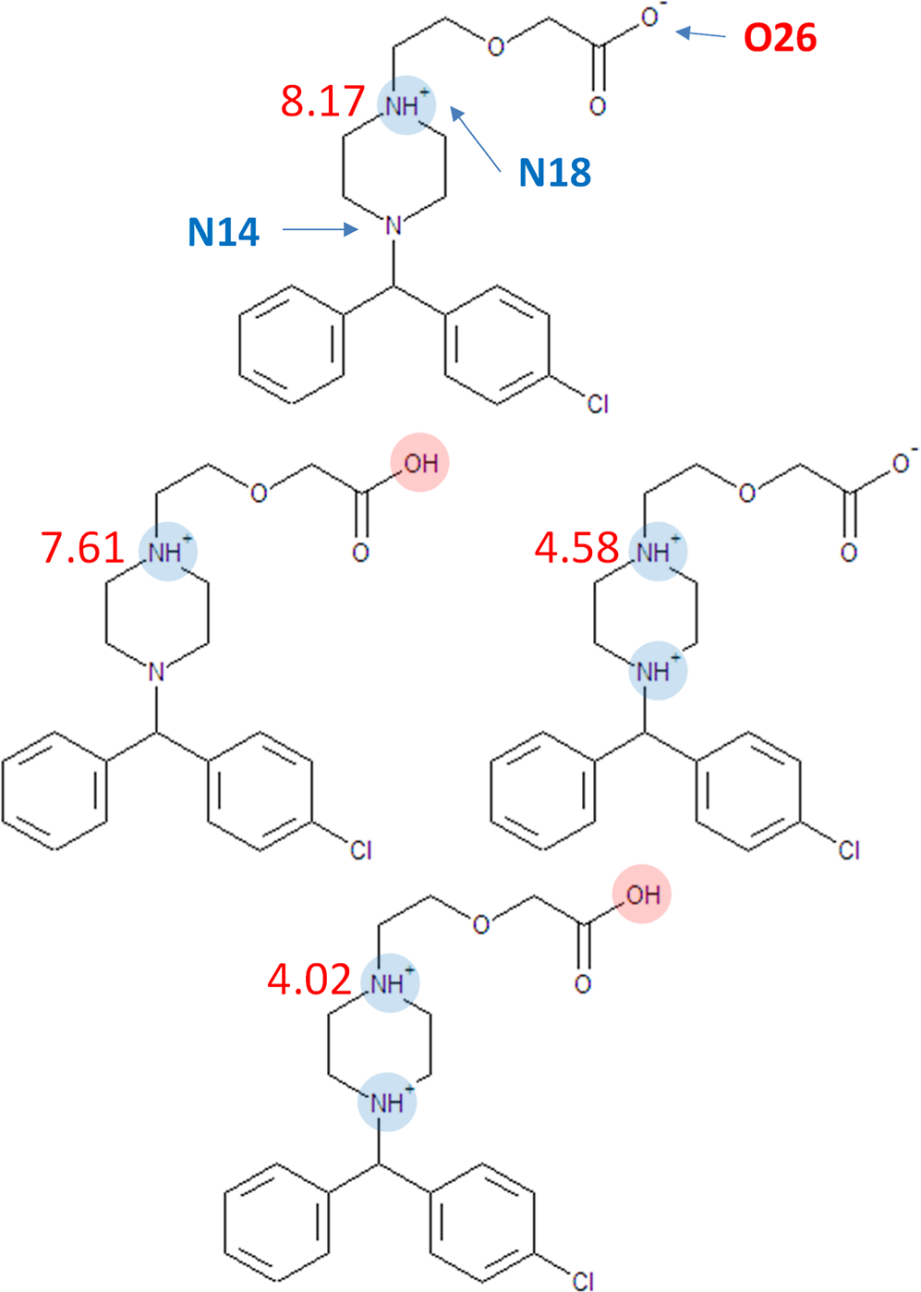
Ionization microconstants of the distal nitrogen in cetirizine,^9^ labeled “N18”, in all microstates where this nitrogen is protonated.

In contrast to macroconstants, microconstants are properties of individual groups. The problem is that microconstants are also functions of the molecular environment. For example, basicity of the distal nitrogen in cetirizine strongly depends on the protonation states of the other two groups (Figure 4). Therefore, questions like “is this amine group strongly or weakly basic?” cannot be answered by microconstants alone, since the answer must be the unsatisfying “it depends”.

An average microconstant, on the other hand, might provide an answer. A careful comparison of Figures 2 and 4 provides an idea on how to properly calculate such average. For the distal amine group, there are four microstates where this group is protonated and the other four where this group is deprotonated (Figures 4 and S1 in Supporting Information 3). One can now imagine a formal “dissociation reaction” where a population of the four protonated microstates loses a proton, yielding a population of the four deprotonated microstates. An “equilibrium constant-like” expression which we named the averaged single-proton acidity (ASPA) for a given ionizable group G is thus defined by the following
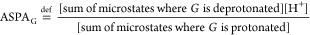
1

Square brackets indicate molar concentrations. The “sum of microstates” is an abbreviation for the sum of respective molar concentrations at a given pH. Because the numerator and denominator in the above equation represent mixtures of different formal charge states, ASPA is not a constant, but a function of pH. To the best of our knowledge, the concept of ASPA is novel.

Another even more useful concept, laboriously derived by protein scientists from statistical thermodynamics considerations and referred to as “site titration curve”,^15,16^ has been adopted by us to the world of small molecules and labeled by averaged site protonation (ASP)
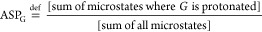
2

The above equation represents fraction protonated of a given, specific ionizable group G. ASP also varies with pH between 0 and the number of dissociable protons on G (usually 1).

Finally, an individual ionizable group property that is a pH-independent constant is called the single-proton midpoint by us and assigned an intuitive symbol of “p*K*_50_”
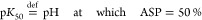
3

The single-proton midpoint corresponds to an intrinsic “group p*K*_a_”. This statement can be supported on statistical thermodynamics grounds.^15,17,18^ Instead of a series of complex equations, we would like to resort to a simple analogy. Dissociation constant, *K*_a_, of a monoprotic acid HA is expressed by
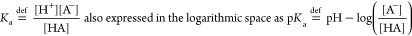
4

The fraction protonated for the monoprotic acid is given by
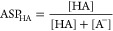
5

At its titration midpoint, we have ASP_HA_ = 50%, leading to the [HA] = [A^–^] equality, thus
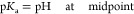
6

Our analogy leads to one more useful relationship. Equations 4 and 5 put together lead to the explicit pH dependence for the fraction protonated of the monoprotic acid
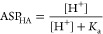
7In the general, multiprotic case, the following relationship between ASPA and ASP of the same group G can be shown as
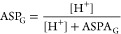
8

Although this equation is reminiscent of eq 7 where ASPA appears instead of the *K*_a_, we would like to underline the pH dependence of ASPA, unlike in the monoprotic case.

The analogy between p*K*_50_ (group) and p*K*_a_ (monoprotic acid) is understood by the virtue of similarity between eqs 3 and 6.

All of the above equations and principles were implemented in our computer program ADMET Predictor in 2012 as an extension to the S+p*K*_a_ predictive model.^4,19^ The model uses artificial neural network ensembles to predict microconstants for 10 major types of ionizable functional groups (hydroxyacids, amide acids, aliphatic amines, heteroaromatic amines, heteroaromatic acidic NH, thioacids, thione bases, N-oxides, acidic CH, and π-excessive carbobases). The predicted values depend on the input tautomer. More details are published in ref (4). The same software was used to perform all subsequent calculations as well as micro- and macroconstant predictions.

## Results and Discussion

**Figure 5:**
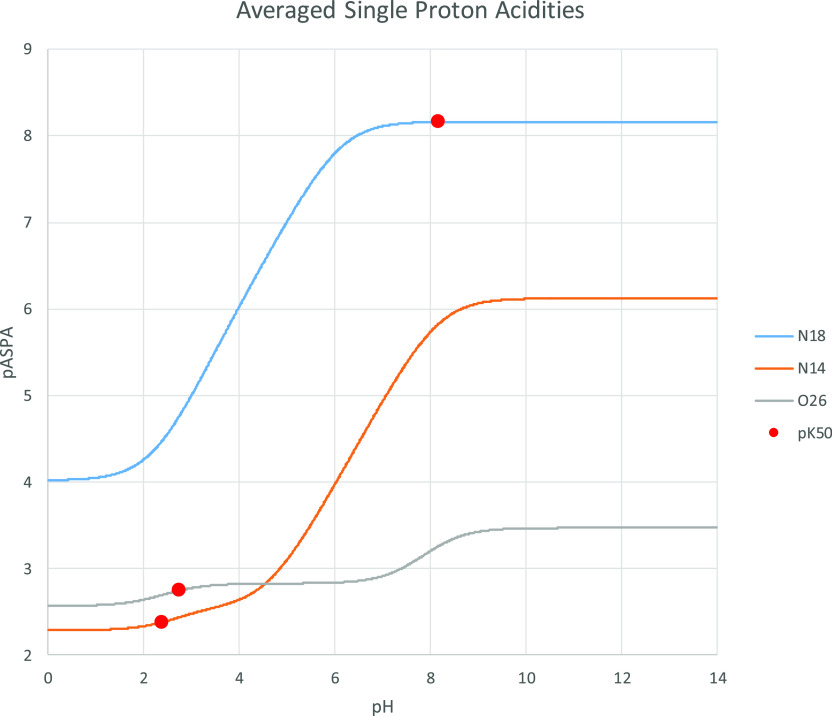
Negative logarithm of the ASPA profiles (pASPA) for cetirizine derived from the experimental microconstants. The orange line labeled “N14” represents the proximal nitrogen, while the blue line labeled “N18” represents the distal nitrogen. Red dots indicate p*K*_50_ values for the respective groups; as expected, these are points of intersection between the ASPA profiles and the identity line (see eqs 3 and 8).

The ionization constant, *K*_a_, as well as ASPA values span a very broad spectrum of magnitudes. Therefore, it is much more convenient to use negative logarithms of these quantities, denoted by p*K*_a_ and pASPA, respectively. The pASPA profiles derived from the measured microconstants for cetirizine^9^ are shown in Figure 5.

The pASPA values increase (i.e., averaged group acidity decreases) with pH for all three ionizable groups. The blue curve representing the distal nitrogen starts at about 4 and plateaus at approximately 8.2 for high pH. This is quite understandable in the view of Figure 4: at low pH, most of the cetirizine molecules are fully protonated and the acidity of the distal nitrogen is at its highest (conversely, its basicity is the lowest) mainly due to repulsive interactions received from the protonated proximal nitrogen. As the pH increases, the population of cetirizine molecules is being gradually deprotonated and average acidity (i.e., average tendency to deprotonate) of the distal nitrogen gradually decreases (its basicity increases) up to the value 8.17, representing its microconstant in the one-proton macrostate. In fact, a general rule for each ionizable group is that its high pH acidity asymptotes at its one-proton microconstant. Similarly, low pH asymptotes are equal to the respective microconstants at the fully protonated macrostate.

**Figure 6:**
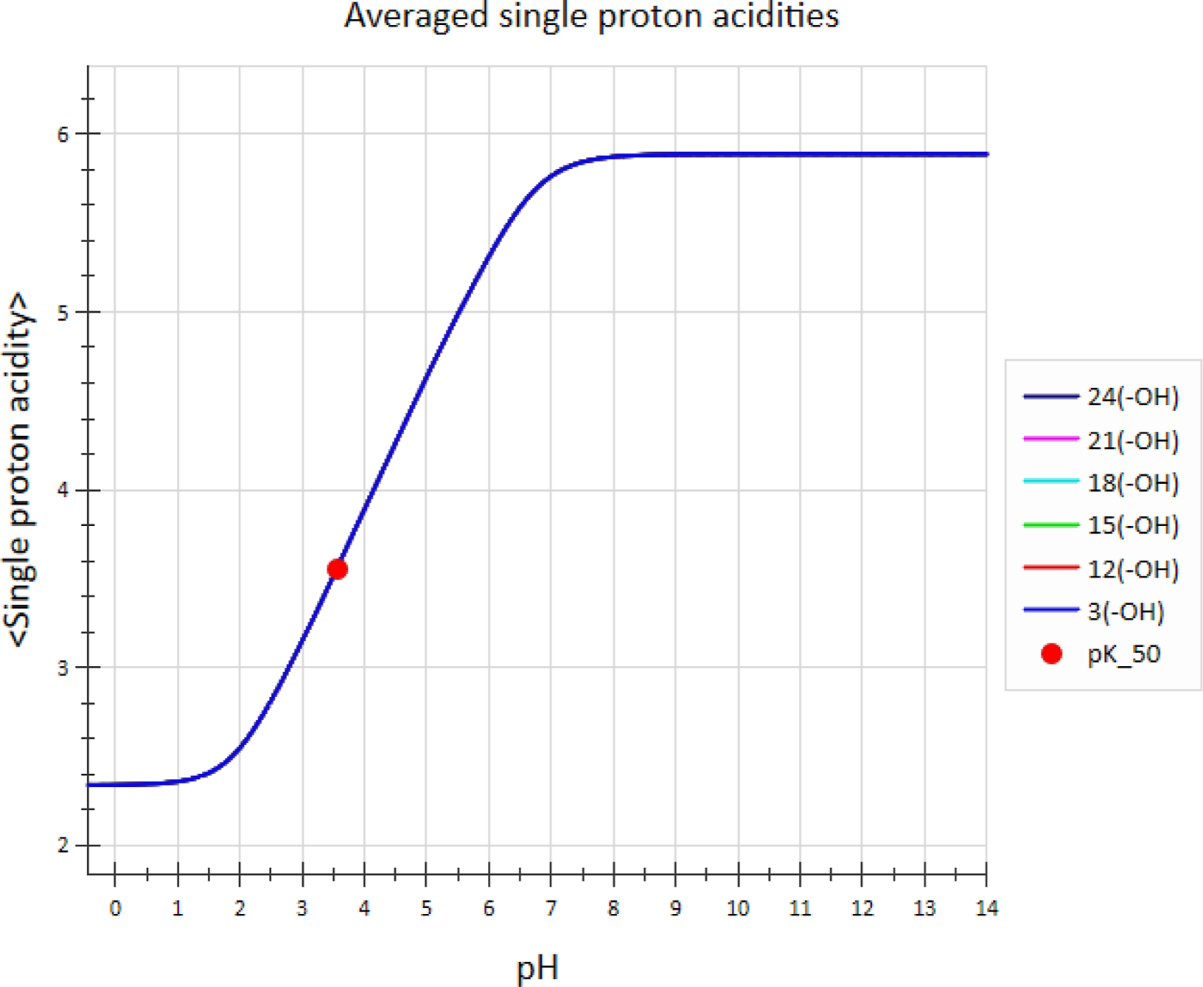
Negative logarithm of the pASPA for mellitic acid derived from microconstants calculated by the ADMET Predictor. Profile curves for all six carboxylic acid groups overlap perfectly. Each carboxyl group’s pASPA varies from 2.34 at low pH to 5.88 at high pH.

What do pASPA curves look like for a system as complex as mellitic acid? Figure 6 reveals that these are quite simple. The pASPA profiles for all six carboxyl groups overlap perfectly, as expected from the molecule’s symmetry. Also expected is the gradual decrease in the individual −COOH acidity with pH, commensurate with the average protonation state of mellitic acid.

**Figure 7:**
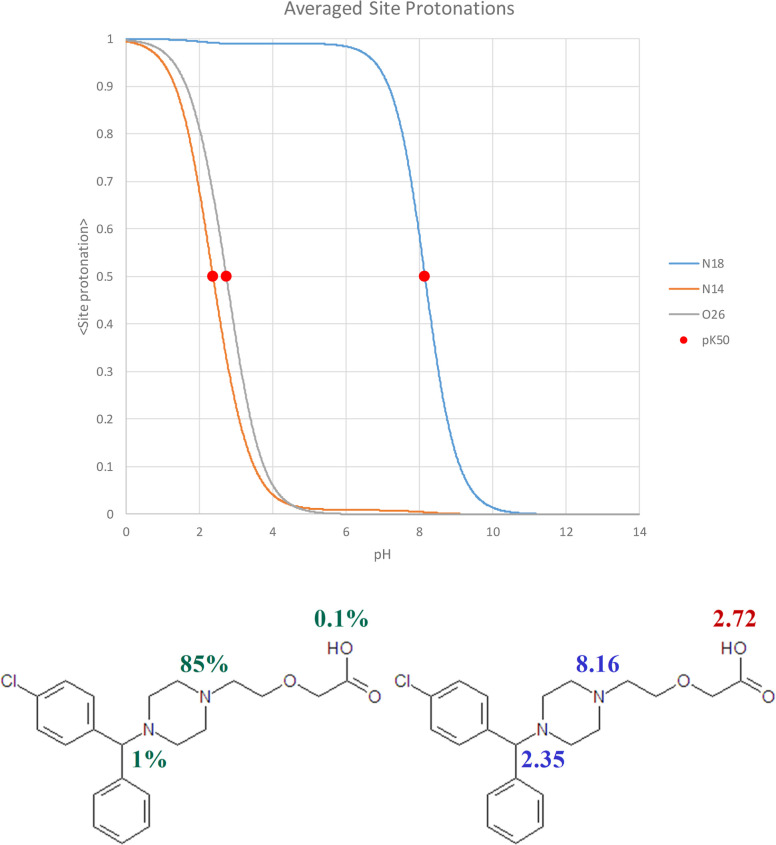
ASP profiles (ASP, a.k.a. fractions protonated) for cetirizine derived from the experimental microconstants. The orange line labeled “N14” represents the proximal nitrogen, while the cyan line labeled “N18” represents the distal nitrogen. Red dots at 0.5 ASP represent p*K*_50_ values. Green percentage values overlapped on the cetirizine structure indicate ASP at pH = 7.4. Blue and red values overlapped on the cetirizine structure are p*K*_50_ for each functional group.

Switching attention to ASP, the ASP profiles obtained from measured microconstants for cetirizine are shown in Figure 7.

As expected, the proximal amine and carboxyl deprotonate quite early at pH ∼ 3. The distal nitrogen stays protonated until much higher pH, and at 7.4, its ASP is 85%. In addition, its ASP midpoint, the p*K*_50_, is at 8.16, very close to the apparent p*K*_a_ of the HCet^0^ macrostate (8.17). This is commensurate with the respective microstate dominance, as discussed above and illustrated in Figure 4. In contrast, the p*K*_50_ values of the proximal N and carboxyl are markedly different from the macroscopic p*K*_a_ they dominate. Thus, the “true” basicity of proximal amine is represented by p*K*_50_ = 2.35, while the experimental H_3_Cet^++^ p*K*_a_ was measured at 2.10. The analogous numbers for the carboxyl group’s acidity are 2.72 and 3.01, respectively. We have observed a general trend for significantly interacting groups: their p*K*_50_ values are closer to each other than the respective apparent p*K*_a_ assigned to protonation macrostates they dominate.

**Figure 8:**
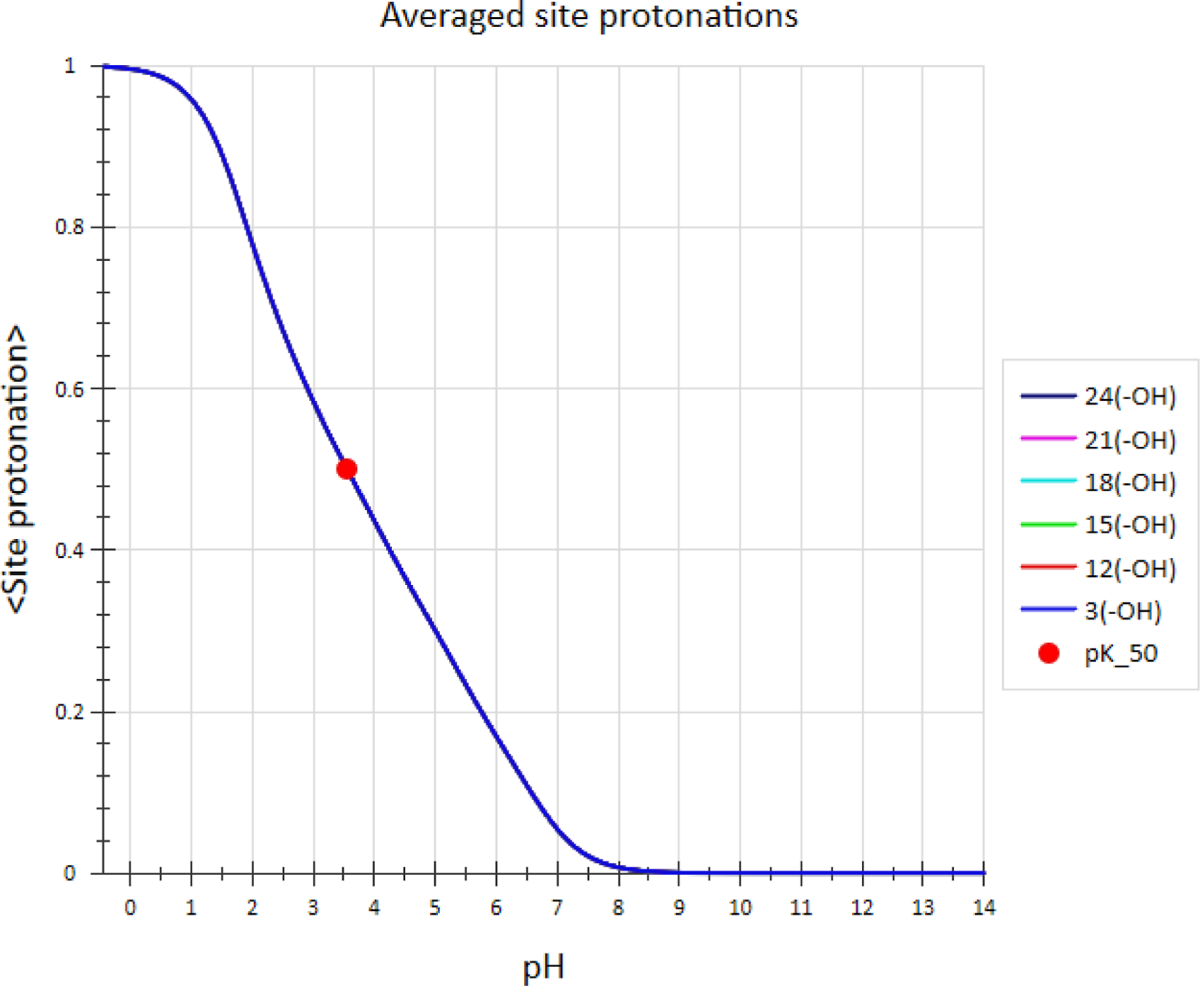
Average site protonation profiles for mellitic acid derived from microconstants calculated by the ADMET Predictor. Profile curves for all six carboxylic acid groups overlap perfectly. Also identical are p*K*_50_ values (red dots) equal to 3.55 for each carboxyl group.

The predicted p*K*_50_ values for carboxyl groups in mellitic acid obtained from the ASP profile shown in Figure 8 are identical and equal to a reasonable value of 3.55. Keep in mind that it is not possible to obtain this measure of group’s acidity from the apparent p*K*_a_ shown in Figure 3.

The pASPA and ASP quantities reduce to the expected simple p*K*_a_ and simple fraction ionized for monoprotic molecules, providing another confirmation of their validity (see Figures S3 and S4 in Supporting Information 3). Additionally, in some relatively rare cases of “pure” ionization macrostates, i.e., the macrostates very strongly dominated by just one microstate, their apparent p*K*_a_ values are numerically very close to the p*K*_50_ of one of the ionizable groups. Such is the case of the HCet^0^ macrostate in cetirizine, but Figure S5 in Supporting Information 3 provides an even more illustrative example. In cefaclor, the acidities of its three functional groups are separated by 4–5 orders of magnitude, resulting in three “pure” macrostates.

How does the ASP relate to the known fraction ionized profile? The latter (a.k.a. Bjerrum plot) is defined as a statistical average of the number of bound ionizable protons dependent on pH. As Figure S6 in Supporting Information 3 illustrates, the molecular fraction ionized profile is a simple sum of the individual ASP plots arranged in the order of decreasing pH.

**Figure 9:**
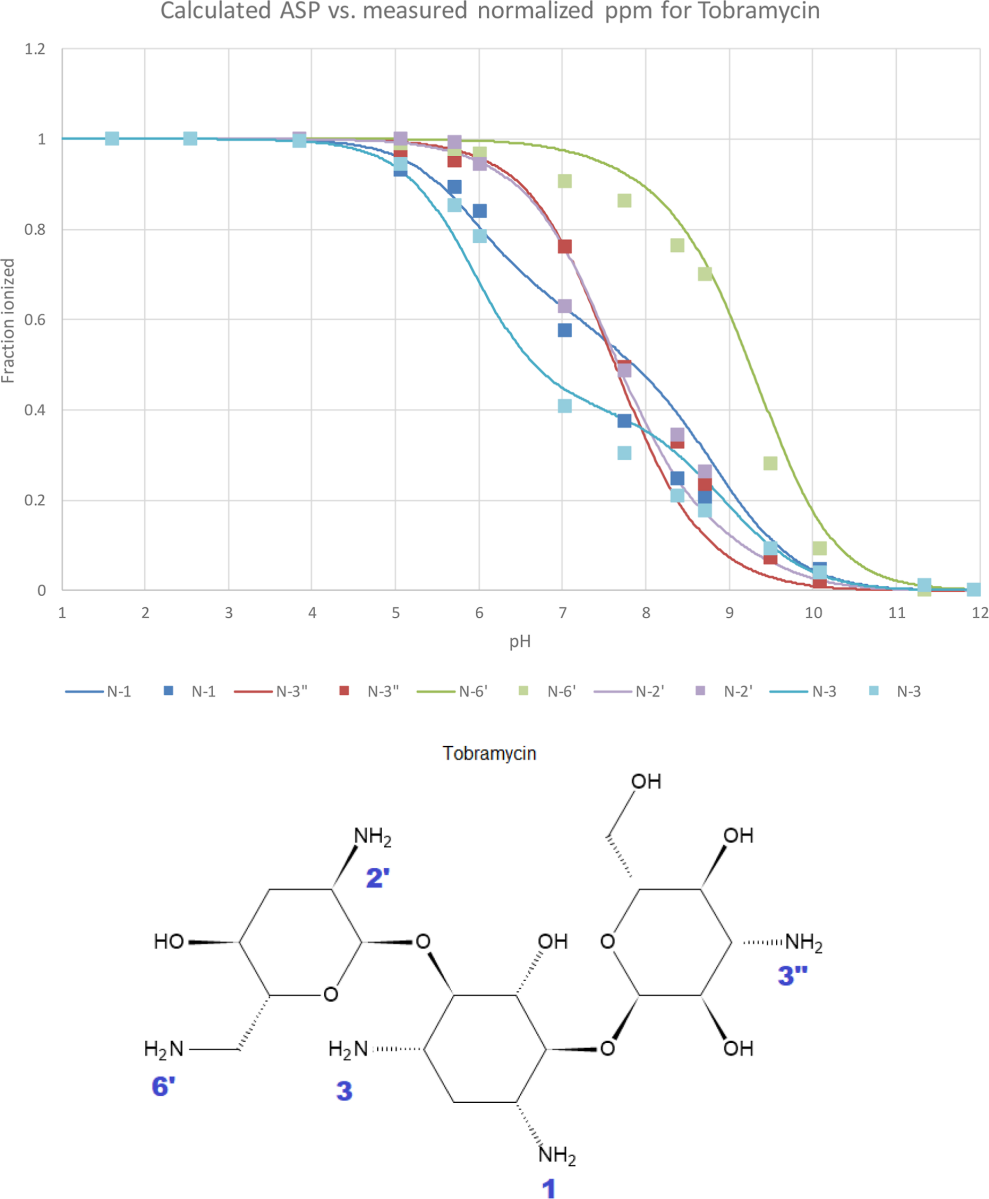
Normalized ^15^N chemical shifts for the five amine groups in tobramycin^21^ (squares) vs predicted^19^ ASP profiles (lines). Amine labels are shown in the structure below.

It is important to note that these predicted values can be verified experimentally with techniques that have atomic resolution and are sufficiently focused on individual ionizable groups. Such is the case for NMR titrations of multiprotic molecules.^20^ Al Khzem et al. performed elaborate heteronuclear multiple-bond correlation titrations of aminoglycosides using ^1^H, ^13^C, and ^15^N chemical shifts.^21−23^ Out of these, the ^15^N spectra were most promising in isolating ionizations of individual aliphatic amine groups. The researchers measured chemical shifts, δ in ppm, of each aliphatic amine group at different pH values and from the resulting δ(pH) plots determined “inflection points of sigmoidal curves”. The thus-obtained “individual p*K*_a_ values not available by potentiometric methods”, in the authors’ language, were in fact approximate p*K*_50_ values of the amines. To better illustrate that such is the case, we have extracted raw ^15^N shifts for all five primary amine groups of tobramycin, an aminoglycoside antibiotic, from Table 4.24 in the Appendix of ref (21). Next, we have normalized these δ values to the [0,1] interval using minimax scaling. In this form, the experimental points are directly comparable to our ASP profiles predicted for tobramycin, as shown in Figure 9. Relevant details can be found in the Excel spreadsheet of Supporting Information 2. The agreement between experimental points and predicted curves is quite good.

Next, we have extracted experimental p*K*_50_ for tobramycin from Table 1 in ref (23) (last row labeled “15N HMBC NMR”) and compared these against our p*K*_50_ predictions.^4,19^ Results shown in Table 1 below once again illustrate good agreement. We have observed similarly good agreement for other aminoglycosides (data provided in Table S1 through S4 in Supporting Information 3).

**Table 1:** Comparison of Observed vs Predicted p*K*_50_ for Tobramycin^a^

	primary amine p*K*_50_ in tobramycin				
method	N-1	N-3	N-2′	N-6′	N-3″
measured^23^	7.55	6.70	7.75	9.10	7.70
predicted^19^	7.85	6.67	7.67	9.25	7.63

a The observed values were taken from the row labeled “^15^N HMBC NMR” in Table 1 in ref (23).

Precise and accurate prediction of individual ionizable p*K*_50_ has important, real-world implications in drug design and development. Relying on the apparent p*K*_a_ “assignments” to a functional group instead may result in costly mistakes. As an example, one of our customers reported the following:

“Our lead compound consisted of a tertiary amine group on one end, a complicated scaffold in the middle, and a substituted phenol ring on the other end. We have measured p*K*_a_ around 9.5 and “assigned” it to the amine. It was crucial to lower the amine’s basicity, and thus, (at great expense) we have synthesized a large number of derivatives with that goal in mind and measured their p*K*_a_. To our surprise, nothing worked—the “amine’s p*K*_a_” did not change or even went up!

**Figure 10:**
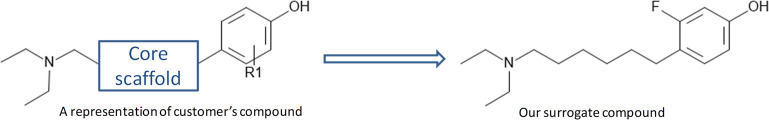
General representation of the customer’s lead compound and our simplified model representing it in calculations. Note that the fluorine substituent does NOT represent R1!

Obviously, we cannot reveal the customer’s proprietary chemical structures, data, or even the derivative pattern. Instead, we have prepared an illustrative example of a made-up surrogate compound, made-up substituents, made-up derivatives, and predicted^4,19^ p*K*_a_. In particular, we have replaced the “complicated scaffold in the middle” by a simple aliphatic chain; see Figure 10.

Table 2 contains predicted p*K*_a_ for the model compound and its derivatives.

**Table 2:**
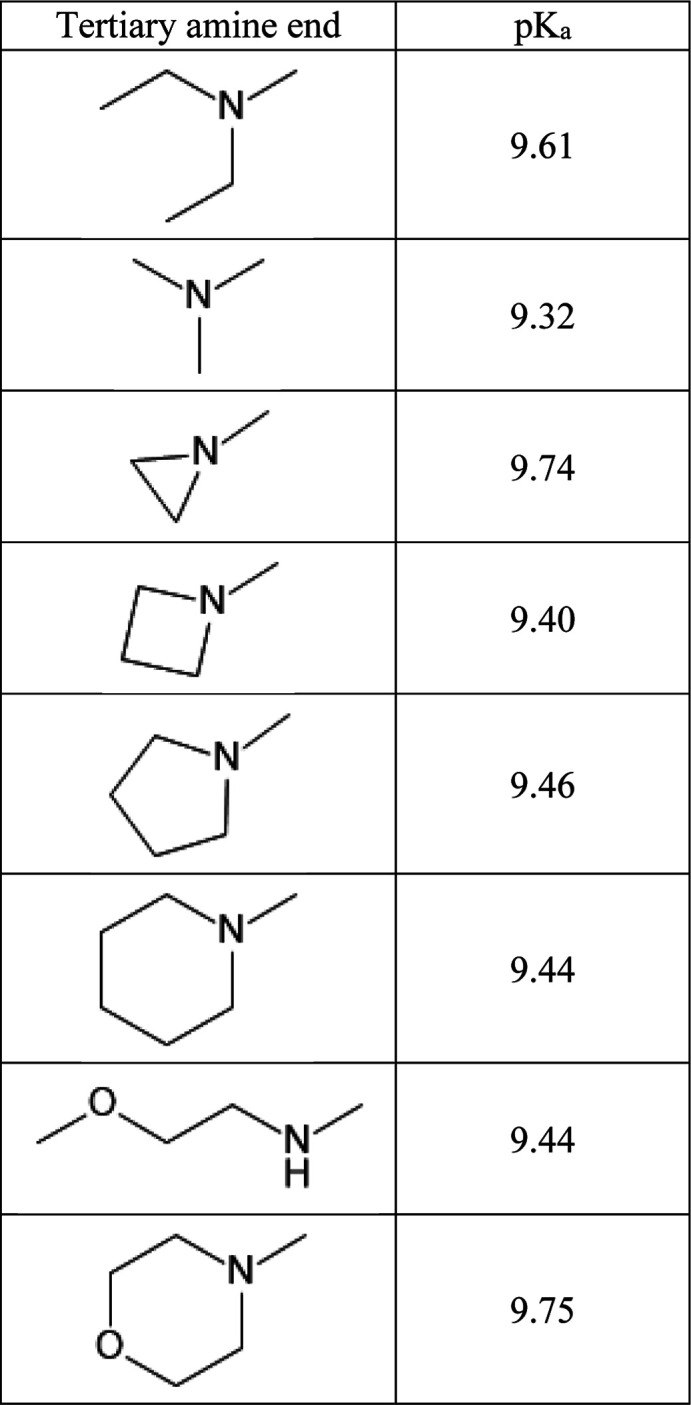
Example of What the Customer May Have Seen in Their Data^a^

a The p*K*_a_ values were predicted for the model compound and its derivatives.

The numbers illustrate a general trend observed by the customer: regardless of the amine substitution, the p*K*_a_ barely changed, in stark contrast to expectations. For example, the strained aziridine ring in the third row should have significantly lowered the amine basicity. Instead, the p*K*_a_ increased by 0.13. The morpholine derivative was expected to lower the amine p*K*_a_ by ∼2 log units, but the apparent “basicity” increased by 0.14.

**Figure 11:**
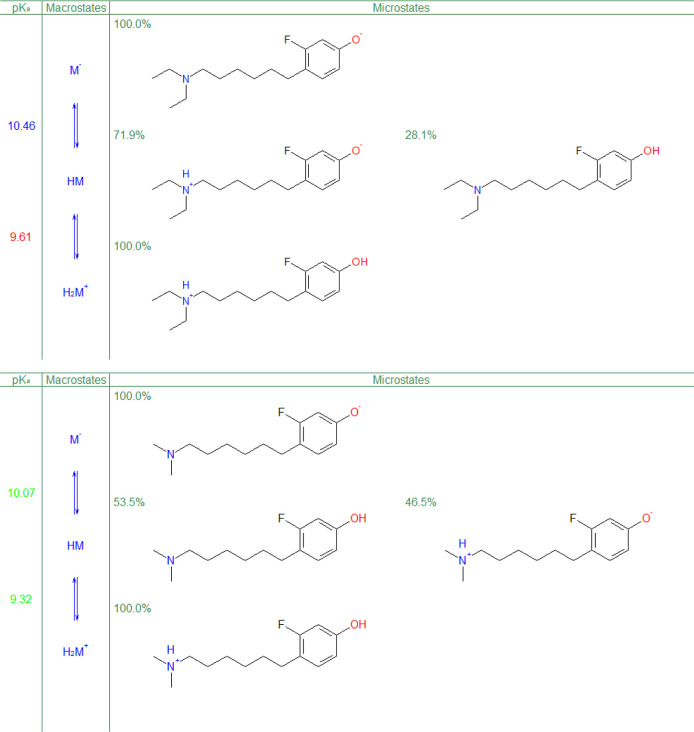
Predicted p*K*_a_ and ionization microstates of the model compound and its dimethyl derivative.

The answer to this behavior is revealed by examining the model compound’s ionization microstates in Figure 11.

The primarily aminic p*K*_a_ (at the 71.9% level) is predicted at 10.46, while the value 9.61 in Table 2 is primarily phenolic. Furthermore, the p*K*_a_ “identity” is not preserved across derivatives—as shown in Figure 11, a higher value of 10.07 is predominantly phenolic, while the lower one may be labeled as aminic. Such an “identity switch” occurs four more times. This illustrates the fallacy of functional group “assignments” using apparent p*K*_a_—the apparent p*K*_a_ is a result of complex interactions of all ionizable groups in a given compound and cannot be assigned to just one group. Indeed, at a 53.5%/46.5% split for the dimethyl derivative, the aminic/phenolic decision cannot be determined as solely acidic or basic.

In contrast, Table 3 reveals that p*K*_50_ values of the tertiary amine change according to expectations (e.g., morpholine p*K*_50_ is markedly lower than the diethyl one).

**Table 3:**
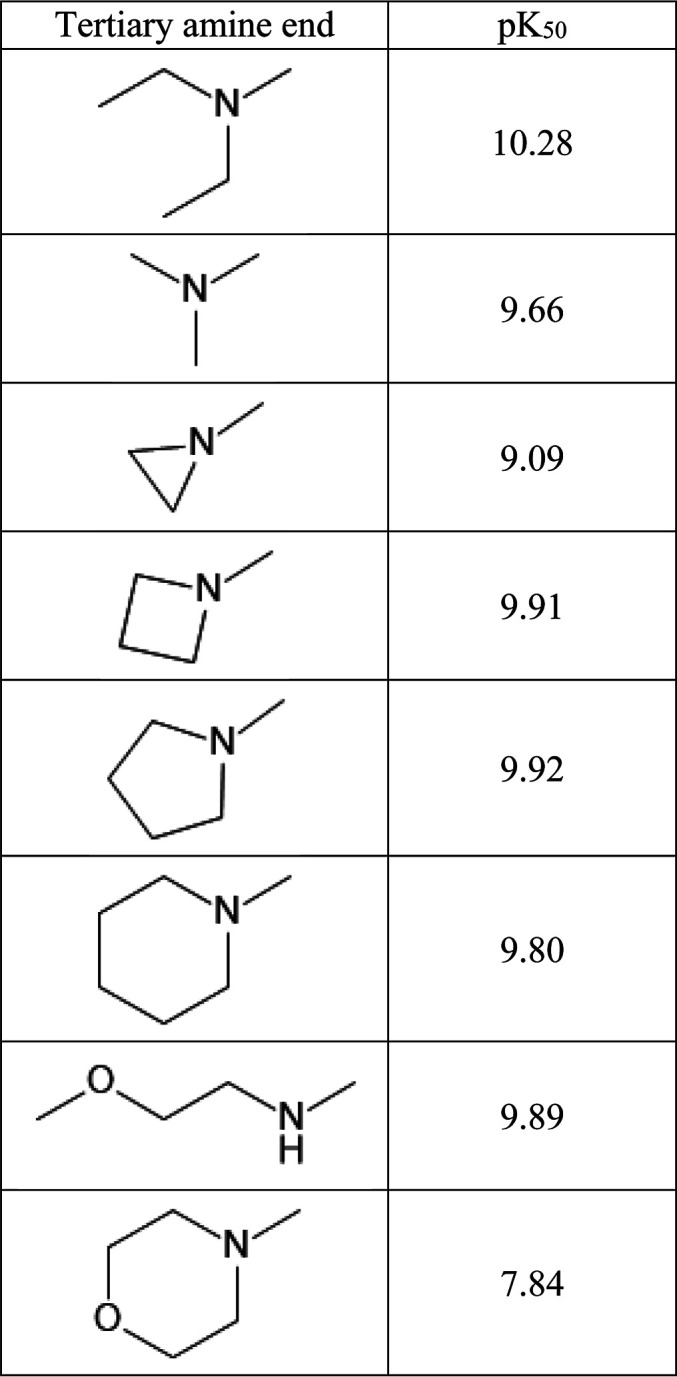
Predicted p*K*_50_ Values for the Tertiary Amine Group in Our Model Series

Having a specific Δp*K*_a_ goal in mind, the customer could then focus on one or two of the most promising derivatives, saving much time and money. Indeed, the customer did perform such suggested computational analysis post mortem and completely agreed with our proposal and conclusions.

The last in our list of examples is Table S5 (Supporting Information 3) containing 24 compounds used in SAMPL6 competition,^24^ their measured macroscopic p*K*_a_, corresponding predicted^19^ p*K*_a_, and predicted p*K*_50_ for major contributing groups.

## Conclusions

Standard titration measurements of multiprotic compounds lead to apparent p*K*_a_ values that represent macroscopic charge state transitions which are statistical averages of the appropriate ionization microstates and as such do not, in general, correspond to quantitative ionization of individual functional groups. Alkhzem et al. note: “potentiometric titration and UV methods have been used to determine ionization constants (p*K*_a_). However, in polyamines, no direct link can be drawn from an individual amino group to a specific p*K*_a_ value (i.e, p*K*_50_) by those techniques.”^22^ Even if an apparent p*K*_a_ happens to be numerically close to one functional group’s p*K*_50_ due to the dominance of a single microstate leading to an “assignment” of the p*K*_a_ to this group, there is no guarantee that such an “assignment” will carry through a congeneric series of related compounds. In fact, as our last example shows, the “assignment” may experience an “identity switch” across the congeneric series due to changes in microstate dominance. This phenomenon may make lead optimization difficult and costly.

The novel concept of ASPA is an intuitive measure of instantaneous acidity/basicity of an ionizable group in a multiprotic molecule. Since the group’s environment (i.e., other ionizable groups present in the molecule) depends on pH, so does its instantaneous acidity/basicity. The pASPA profiles show a general trend of higher acidity/lower basicity at low pH that gradually decreases/increases with growing pH, respectively.

The ASPA led us quite naturally to a previously known^15^ concept of the probability of an ionizable group being protonated (a.k.a. fraction protonated) which we named the ASP. Similar to ASPA, the ASP depends on pH because the group’s environment depends on pH. The ASP profiles usually resemble sigmoid titration curves. It is the midpoint of an ASP profile that provides an unambiguous and pH-independent measure of the true group’s acidity—the p*K*_50_. The influence of other groups on the ASP midpoint is effectively averaged, and thus, the p*K*_50_ carries through congeneric series in an expected manner regardless of microstate dominance shuffling, unlike the apparent p*K*_a_. Moreover, p*K*_50_ values are directly measurable in elaborate NMR titration experiments with atomic resolution. We suggest that p*K*_50_ should replace apparent p*K*_a_ in applications involving acidity of individual functional groups.

Since experimental determination of ionization microconstants and p*K*_50_ is still too expensive for routine molecular design, in our humble opinion, predictive computer modeling will remain the main avenue of obtaining these useful quantities. There are many software programs that predict microconstants via either linear free energy relationships, quantitative structure–property relationships, or ab initio quantum chemical calculations. See refs (2) and (3) for detailed discussion of these methods. Simple source code modifications may tailor any of these programs toward computing p*K*_50_ values as well.

## Supporting Information Available

Predicted microstates of mellitic acid (TIF)
Data used to draw Figure 9 (XLSX)
Illustration of the fundamental concept behind ASPA, predicted p*K*_a_ and microstate probabilities, predicted pASPA profile for acetic acid, predicted p*K*_50_, predicted statistical average, comparison of observed vs predicted p*K*_50_, and measured macroscopic p*K*_a_ (PDF)

## Notes

ADMET Predictor is available for evaluation and academic research purposes free of charge.

## Footnotes

a To avoid confusion, we would like to stress that the following discussion of cetirizine ionization patterns is in its entirety based on measured microconstants. For comparison, predicted p*K*_a_ values for this compound are presented in Figure S2 in Supporting Information 3.

b Although quoted ionization macroconstants for mellitic acid are measured, its microscopic ionization patterns presented in Supporting Information 1 are predicted.

## References

[ref1] Rupp M.; Korner R.; V Tetko I. Predicting the pKa of Small Molecules. Comb. Chem. High Throughput Screening 2011, 14, 307–327. 10.2174/138620711795508403. 21470178

[ref2] Fraczkiewicz R. In silico Prediction of Ionization. In Comprehensive Medicinal Chemistry II; Testa B., van de Waterbeemd H., Eds.; Elsevier: Oxford, United Kingdom, 2006; Vol. 5, p 603.

[ref3] Fraczkiewicz R. In silico Prediction of Ionization. In Reference Module in Chemistry, Molecular Sciences and Chemical Engineering; Reedijk J., Ed.; Elsevier, 2013; Vol. 5.

[ref4] Fraczkiewicz R.; Lobell M.; Göller A. H.; Krenz U.; Schoenneis R.; Clark R. D.; Hillisch A. Best of Both Worlds: Combining Pharma Data and State of the Art Modeling Technology To Improve in Silico pKa Prediction. J. Chem. Inf. Model. 2015, 55, 389–397. 10.1021/ci500585w. 25514239

[ref5] Bjerrum N. Dissoziationkonstanten von Mehrbasischen Sauren und ihre Anwendung zur Berechnung Molekular Dimensionen. Z. Phys. Chem., Stoechiom. Verwandtschaftsl. 1923, 106U, 219–242. 10.1515/zpch-1923-10615.

[ref6] Borkovec M.; Brynda M.; Koper G. J. M.; Spiess B. Resolution of Microscopic Protonation Mechanisms in Polyprotic Molecules. Chimia 2002, 56, 695 10.2533/000942902777679911.

[ref7] Noszal B. Microspeciation of Polypeptides. J. Phys. Chem. 1986, 90, 6345–6349. 10.1021/j100281a056.

[ref8] Szakács Z.; Noszál B. Protonation Microequilibrium Treatment of Polybasic Compounds with Any Possible Symmetry. J. Math. Chem. 1999, 26, 139 10.1023/A:1019133927929.

[ref9] Marosi A.; Kovacs Z.; Beni S.; Kokosi J.; Noszal B. Triprotic Acid-Base Microequilibria and Pharmacokinetic Sequelae of Cetirizine. Eur. J. Pharm. Sci. 2009, 37, 321–328. 10.1016/j.ejps.2009.03.001. 19491022

[ref10] Mernissi-Arifi K.; Schmitt L.; Schlewer G.; Spiess B. Complete Resolution of the Microscopic Protonation Equilibria of D-Myo-Inositol 1,2,6-Tris(phosphate) and Related Compounds by 31P NMR and Potentiometry. Anal. Chem. 1995, 67, 2567–2574. 10.1021/ac00111a012.

[ref11] Peinhardt G.; Wiese M. Microionization Constants: Novel Approach for the Determination of the Zwitterionic Equilibrium of Hydroxyphenylalkylamines by Photometric Titration. Int. J. Pharm. 2001, 215, 83–89. 10.1016/s0378-5173(00)00673-6. 11250094

[ref12] Szakacs Z.; Kraszni M.; Noszal B. Determination of Microscopic Acid-Base Parameters from NMR-pH Titrations. Anal. Bioanal. Chem. 2004, 378, 1428–1448. 10.1007/s00216-003-2390-3. 15214406

[ref13] Tarn K. Y. Multiwavelength Spectrophotometric Determination of Acid Dissociation Constants. Part VI. Deconvolution of Binary Mixtures of Ionizable Compounds. Anal. Lett. 2000, 33, 145 10.1080/00032710008543043.

[ref14] Maxwell W. R.; Partington J. R. The Dissociation Constants of Some Polybasic Acids. Trans. Faraday Soc. 1935, 31, 922 10.1039/tf9353100922.

[ref15] Bashford D.; Karplus M. pKa’s of Ionizable Groups in Proteins: Atomic Detail from a Continuum Electrostatic Model. Biochemistry 1990, 29, 10219–10225. 10.1021/bi00496a010. 2271649

[ref16] Beroza P.; Fredkin D. R.; Okamura M. Y.; Feher G. Protonation of Interacting Residues in a Protein by a Monte Carlo Method: Application to Lysozyme and the Photosynthetic Reaction Center of Rhodobacter sphaeroides. Proc. Natl. Acad. Sci. U.S.A. 1991, 88, 5804–5808. 10.1073/pnas.88.13.5804. 2062860 PMC51966

[ref17] Bashford D. Macroscopic Electrostatic Models for Protonation States in Proteins. Front. Biosci. 2004, 9, 1082 10.2741/1187. 14977531

[ref18] Bashford D.; Karplus M. Multiple-Site Titration Curves of Proteins: An Analysis of Exact and Approximate Methods for Their Calculation. J. Phys. Chem. 1991, 95, 9556–9561. 10.1021/j100176a093.

[ref19] ADMET Predictor(R) v 10.4; Simulations Plus, Inc.: Lancaster, CA, USA, 2022.

[ref20] Blagbrough I. S.; Metwally A. A.; Geall A. J. Measurement of Polyamine pKa Values. In Polyamines: Methods and Protocols; Pegg A. E., Casero J., Robert A., Eds.; Springer Science + Business Media, 2011; p 493. 10.1007/978-1-61779-034-8_32 21318895

[ref21] Al Khzem A. H. Medicinal Chemistry of Aminoglycosides. Master Thesis; University of Bath, 2019.

[ref22] Alkhzem A. H.; Woodman T. J.; Blagbrough I. S. Multinuclear Nuclear Magnetic Resonance Spectroscopy Is Used to Determine Rapidly and Accurately the Individual pKa Values of 2-Deoxystreptamine, Neamine, Neomycin, Paromomycin, and Streptomycin. ACS Omega 2021, 6, 2824–2835. 10.1021/acsomega.0c05138. 33553900 PMC7860104

[ref23] Alkhzem A. H.; Woodman T. J.; Blagbrough I. S. Individual pKa Values of Tobramycin, Kanamycin B, Amikacin, Sisomicin, and Netilmicin Determined by Multinuclear NMR Spectroscopy. ACS Omega 2020, 5, 21094–21103. 10.1021/acsomega.0c02744. 32875246 PMC7450637

[ref24] Işık M.; Rustenburg A. S.; Rizzi A.; Gunner M. R.; Mobley D. L.; Chodera J. D. Overview of the SAMPL6 pKa challenge: evaluating small molecule microscopic and macroscopic pKa predictions. J. Comput.-Aided Mol. Des. 2021, 35, 131–166. 10.1007/s10822-020-00362-6. 33394238 PMC7904668

