# Supplementary figures and images for: pK50—A Rigorous Indicator of Individual Functional Group Acidity/Basicity in Multiprotic Compounds
Source: J Chem Inf Model. 2023 Apr 27;63(10):3198–208. doi: 10.1021/acs.jcim.3c00187 (PMC10207274; doi:10.1021/acs.jcim.3c00187)

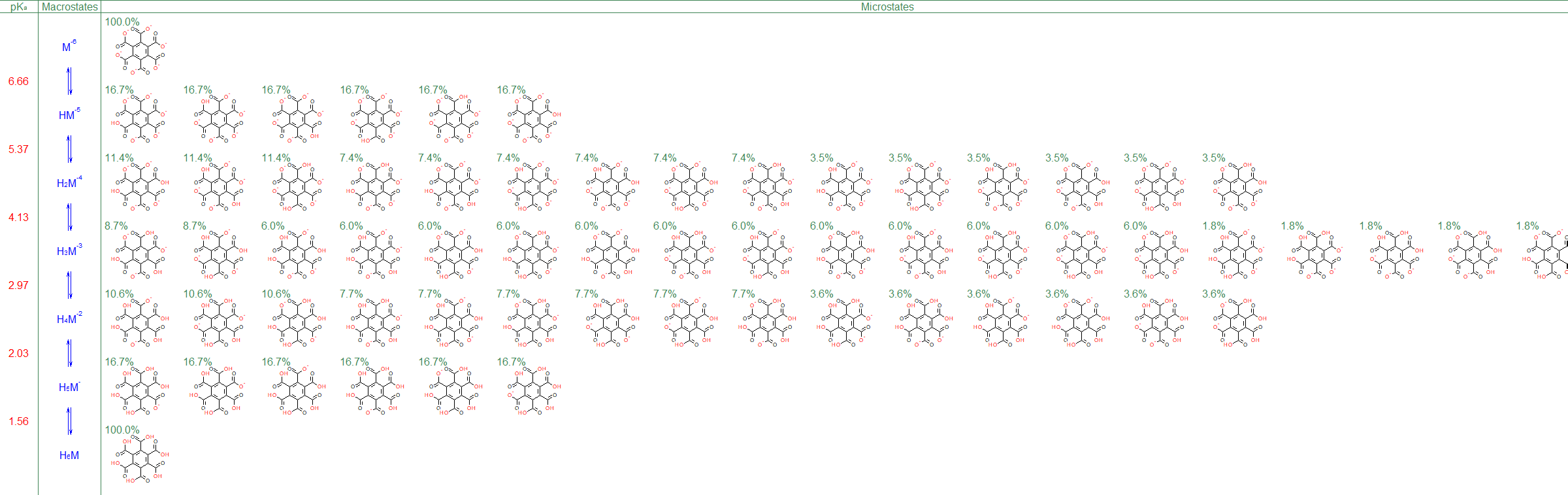

Supplement: Supplementary file 1 — ci3c00187_si_001.tif [file ci3c00187_si_001.tif]
