# Supplementary material for: pK50—A Rigorous Indicator of Individual Functional Group Acidity/Basicity in Multiprotic Compounds
Source: J Chem Inf Model. 2023 Apr 27;63(10):3198–208. doi: 10.1021/acs.jcim.3c00187 (PMC10207274; doi:10.1021/acs.jcim.3c00187)
Supplement: Supplementary file 3 — ci3c00187_si_003.pdf [file ci3c00187_si_003.pdf]

Supplementary Information 3

pK_{50} - a Rigorous Indicator of Individual Functional Group Acidity/Basicity in Multiprotic Compounds.

Robert Fraczkiewicz, Marvin Waldman

Simulations Plus, Inc. 42505 10th Street West, Lancaster, CA 93534, USA.

E-mail: robert.fraczkiewicz@simulations-plus.com

Figure S 1. Illustration of the fundamental concept behind ASPA: there are exactly one half of microstates with the distal amine protonated (blue frame) and one half where it is deprotonated (red frame). All numbers are calculated from measured microconstants.[1]

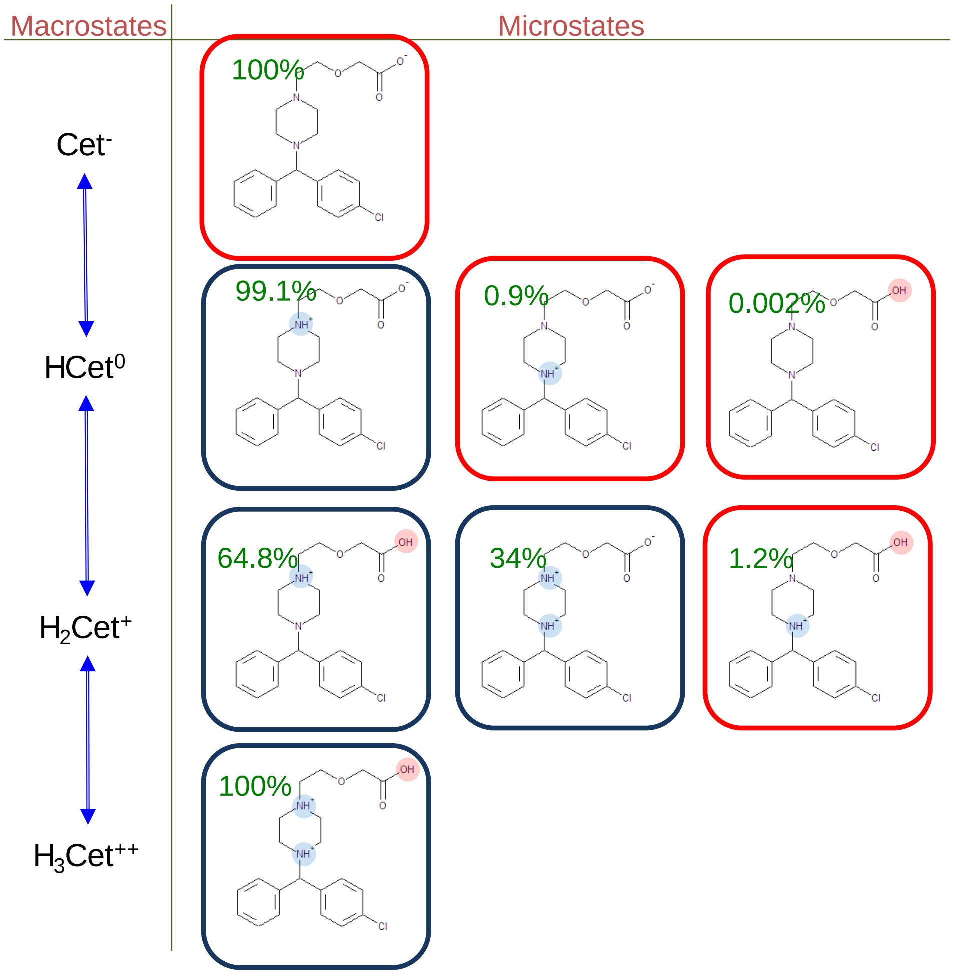

Figure S 2. Predicted[2] pK_a and microstate probabilities for cetirizine.

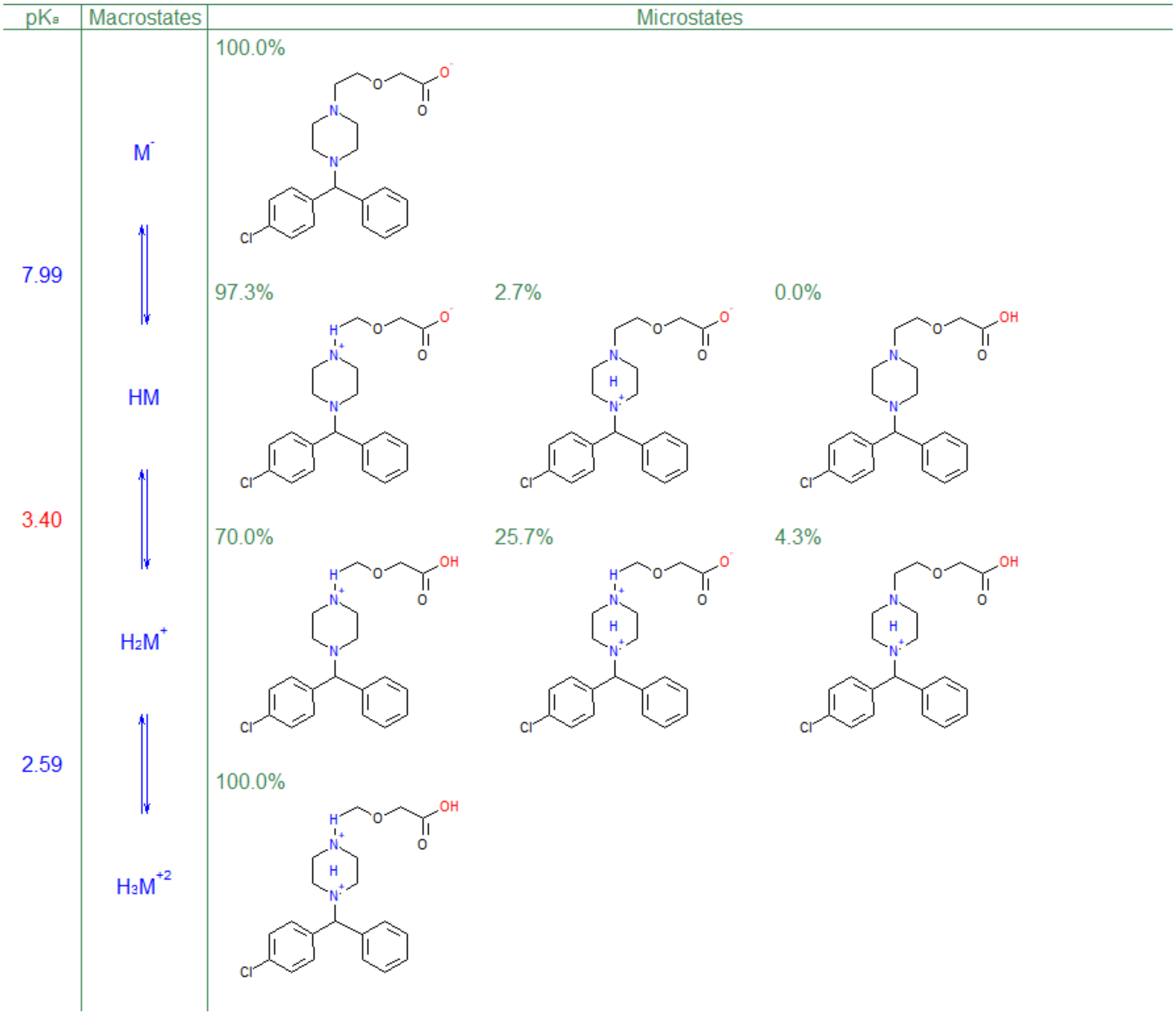

Figure S 3. The predicted[2] pASPA profile for acetic acid is flat, as expected.

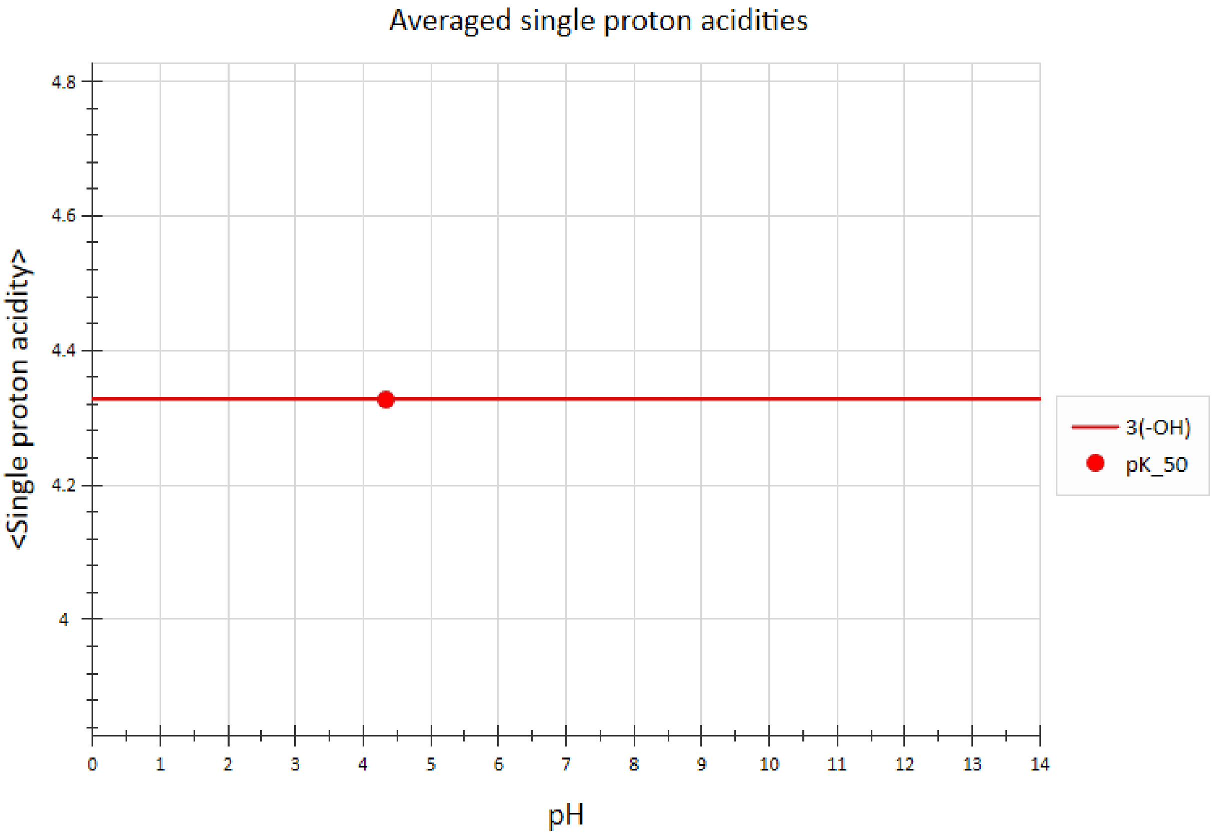

Figure S 4. The predicted[2] ASP profile for acetic acid resembles a monoprotic acid titration curve, as expected.

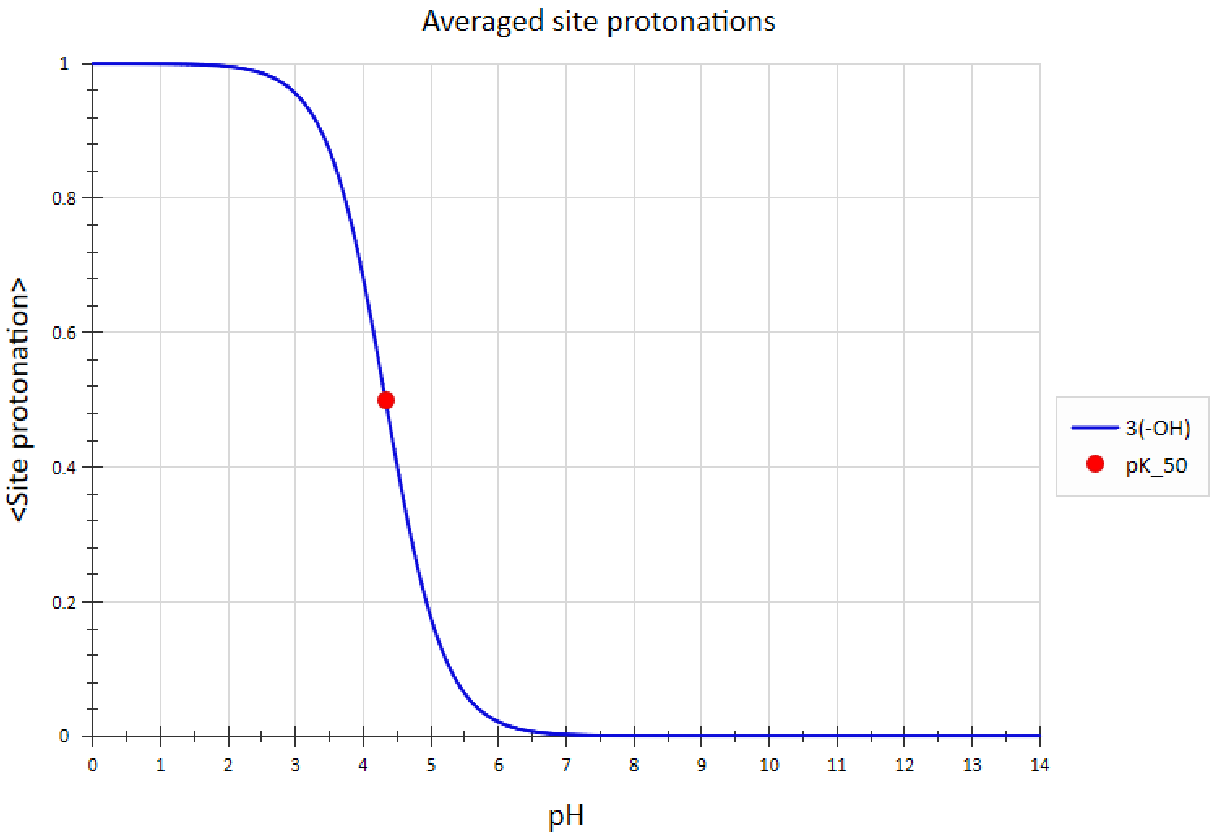

Figure S 5. In the case of very well separated functional groups in terms of their acidity, the predicted[2] pK_{50} coincide with corresponding apparent pK_a. In the example below, acidities of cefaclor's groups are 4-5 orders of magnitude apart from each other. Consequently, each ionization macrostate is strongly dominated by a single microstate and its pK_a is almost equal to the relevant pK_{50}.

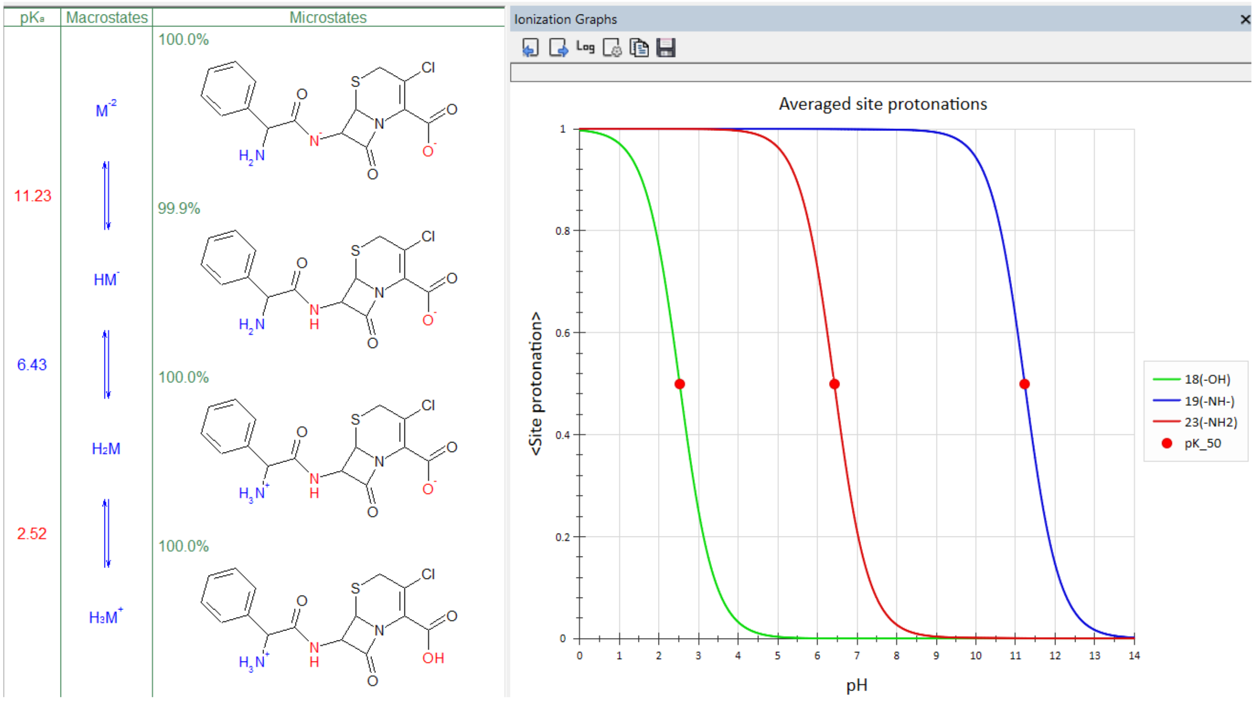

Cefaclor

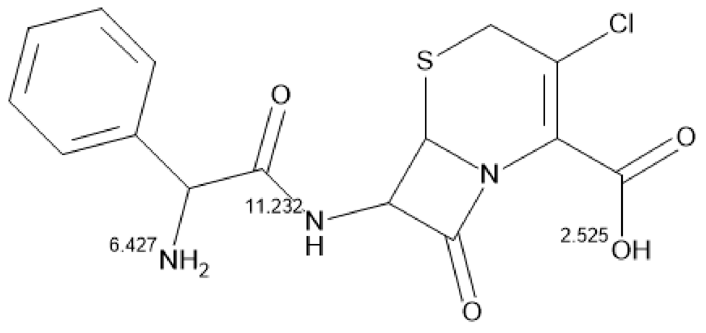

pK_50 Values

Figure S 6. Predicted[2] statistical average of the number of bound ionizable protons as a function of pH (a.k.a. Bjerrum plot; a.k.a. fraction ionized plot) for cefaclor is a simple sum of its three individual group ASP profiles. See Figure S 5 for comparison.

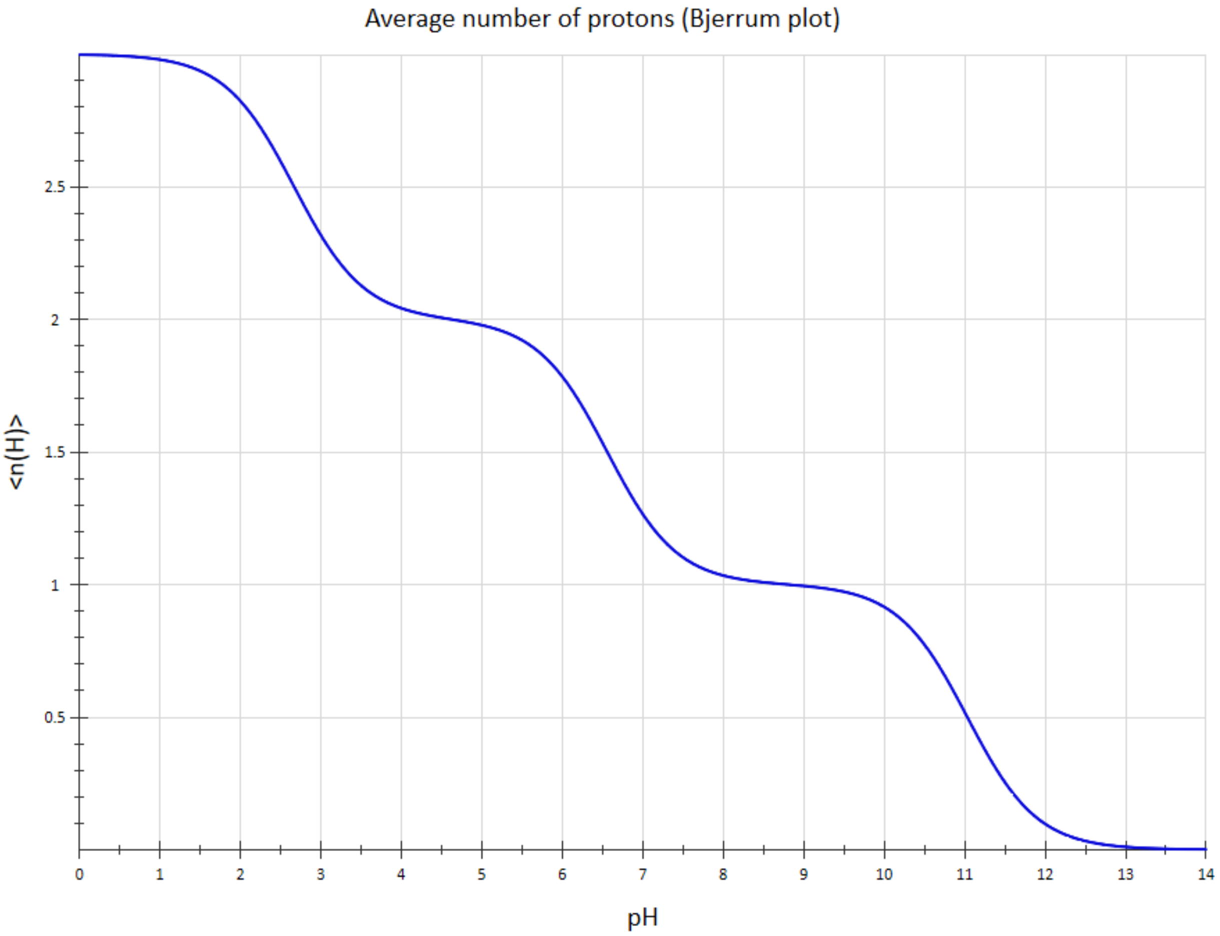

Table S 1. Comparison of observed vs. predicted[2] pK_{50} for kanamycin B. The observed values were taken from the row labeled "^{15}N HMBC NMR" in Table 2 in Ref. [3].

	Primary amine pK_{50} in kanamycin B				
Method	N-1	N-3	N-2'	N-6'	N-3"
Measured[3]	8.05	6.85	7.35	8.90	7.65
Predicted[2]	7.84	6.99	7.07	9.21	7.68

Table S 2. Comparison of observed vs. predicted[2] pK_{50} for amikacin. The observed values were taken from the row labeled "^{15}N HMBC NMR" in Table 3 in Ref. [3].

	Primary amine pK_{50} in amikacin			
Method	N-3	N-6'	N-3"	N-4"
Measured[3]	7.60	8.80	8.10	9.90
Predicted[2]	8.06	9.21	7.51	9.88

Table S 3. Comparison of observed vs. predicted[2] pK_{50} for sisomicin. The observed values were taken from the row labeled "^{15}N HMBC NMR" in Table 4 in Ref. [3].

	Primary amine pK_{50} in sisomicin				
Method	N-1	N-3	N-2'	N-6'	N-3"
Measured[3]	7.41	6.24	8.05	9.29	8.55
Predicted[2]	7.56	6.61	7.31	8.80	8.09

Table S 4. Comparison of observed vs. predicted[2] pK_{50} for netilmicin. The observed values were taken from the row labeled "^{15}N HMBC NMR" in Table 5 in Ref. [3].

	Primary amine pK_{50} in netilmicin				
Method	N-1	N-3	N-2'	N-6'	N-3"
Measured[3]	8.20	6.51	8.23	9.37	8.45
Predicted[2]	8.50	6.15	7.43	8.78	8.01

Table S 5. Measured macroscopic pK_a, corresponding predicted[2] macroscopic pK_a, and predicted main contributor pK_{50} for 24 compounds used in SAMPL6 blind competition[4] of predictive pK_a models. Simulations Plus participated in this competition under the "*hdiyq*" and "*gyuhx*" identifiers taking 1st place among empirical models.

Compound ID	Structure	Measured pK_a	Predicted pK_a	Predicted pK_{50}
SM01	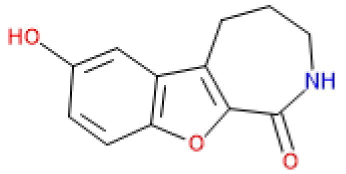	9.53	9.36	9.36 (OH)
SM02	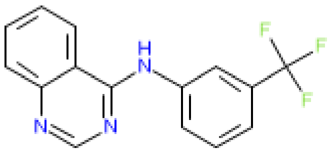	5.03	4.55	4.52 (ring N, *para* to NH)
SM03	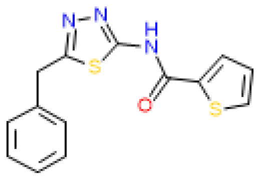	7.02	8.97	8.97 (amide NH)
SM04	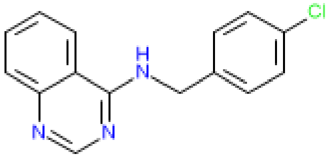	6.02	5.36	5.35 (ring N, *para* to NH)
SM05	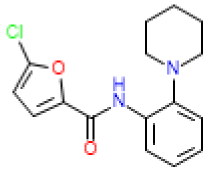	4.59	4.06	4.06 (tertiary N)

SM06	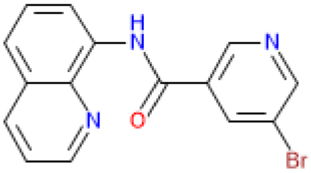	11.74, 3.03	10.72, 3.41	10.72 (amide NH)<br><br>3.4 (quinoline N)
SM07	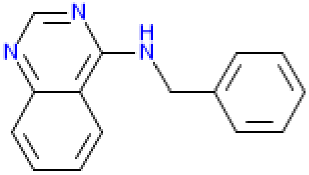	6.08	5.59	5.58 (ring N, *para* to NH)
SM08	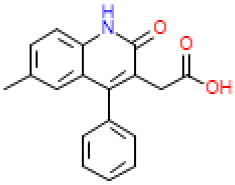	4.22	4.11	4.11 (carboxyl)
SM09	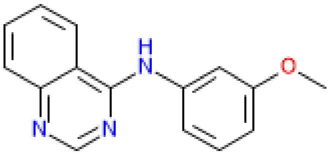	5.37	4.51	4.48 (ring N, *para* to NH)
SM10	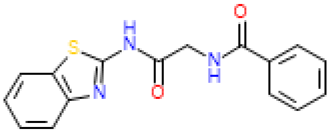	9.02	10.24	10.28 (amide NH, proximal to benzothiazoline)
SM11	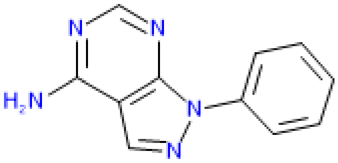	3.89	3.93	3.73 (ring N, *ortho* to NH_2)
SM12	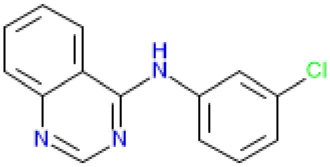	5.28	4.25	4.23 (ring N, *para* to NH)

SM13	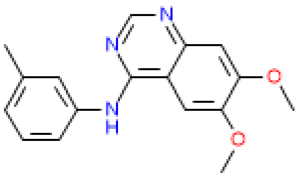	5.77	4.85	4.81 (ring N, *para* to NH)
SM14	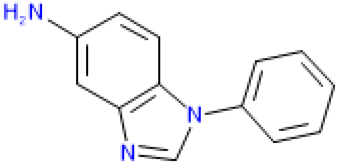	5.3, 2.58	5.35, 3.4	5.17 (ring N)<br><br>3.58 (NH_2)
SM15	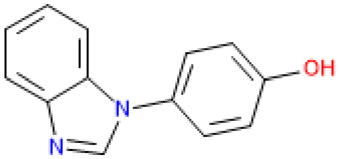	8.94, 4.7	9.12, 4.35	9.12 (OH)<br><br>4.35 (ring N)
SM16	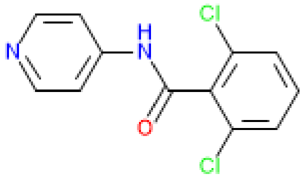	10.65, 5.37	10.79, 5.21	10.79 (amide NH)<br><br>5.21 (ring N)
SM17	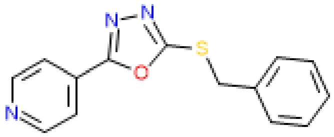	3.16	3.78	3.78 (pyridine N)
SM18	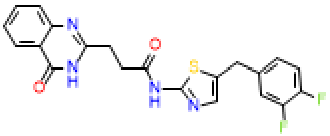	11.02, 9.58, 2.15	10.23, 9.21, 2.72	9.86 (amide NH, chain)<br><br>9.57 (amide NH, ring)<br><br>2.43 (thiazole N)
SM19	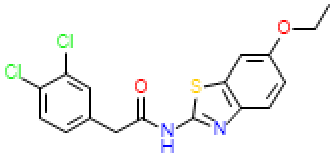	9.56	9.94	9.94 (amide NH)

SM20	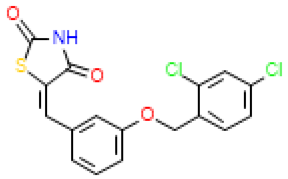	5.7	6.77	6.77 (amide NH)
SM21	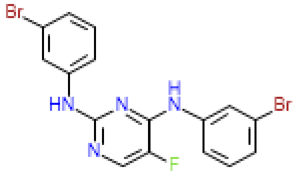	4.1	2.92	2.78 (ring N, *para* to NH)
SM22	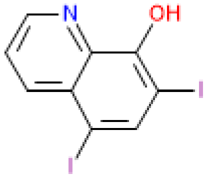	7.43, 2.4	8.17, 2.13	8.17 (OH)<br><br>2.13 (ring N)
SM23	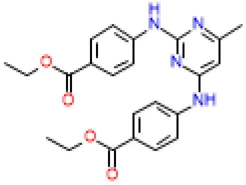	5.45	5.37	5.36 (ring N, *para* to NH)
SM24	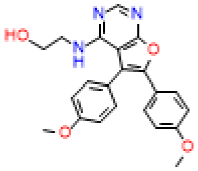	2.6	3.55	2.27 (ring N, *para* to NH)

References

(1) Marosi, A.; Kovacs, Z.; Beni, S.; Kokosi, J.; Noszal, B. Triprotic Acid-Base Microequilibria and Pharmacokinetic Sequelae of Cetirizine. *Eur. J. Pharm. Sci.* **2009**, *37*, 321.

(2) ADMET Predictor(R) *v 10.4*; Simulations Plus, Inc.: Lancaster, CA, USA, **2022**

(3) Alkhzem, A. H.; Woodman, T. J.; Blagbrough, I. S. Individual pKa Values of Tobramycin, Kanamycin B, Amikacin, Sisomicin, and Netilmicin Determined by Multinuclear NMR Spectroscopy. *ACS Omega* **2020**, *5*, 21094.

(4) Işık, M.; Rustenburg, A. S.; Rizzi, A.; Gunner, M. R.; Mobley, D. L.; Chodera, J. D. Overview of the SAMPL6 pKa challenge: evaluating small molecule microscopic and macroscopic pKa predictions. *J. Comput.-Aided Mol. Des.* **2021**, *35*, 131.
